# Occupational survey-based evidence of health status and welfare problems of workers with pneumoconiosis in China

**DOI:** 10.3389/fpubh.2023.1142161

**Published:** 2023-08-31

**Authors:** Wenxiu Hu, Wei-Ning Wu, Qingmei Qiao

**Affiliations:** ^1^Centre for Population and Development Policy Studies, Fudan University, Shanghai, China; ^2^Institute of Public Affairs Management, National Sun Yat-sen University, Kaohsiung, Taiwan; ^3^Department of Social Security, School of Labor and Human Resources, Renmin University of China, Beijing, China

**Keywords:** risk, social welfare policy, environmental justice, social sustainability, occupational health

## Abstract

**Background:**

Pneumoconiosis is the most dangerous occupational disease in China. According to unofficial records, nearly million migrant workers were affected by pneumoconiosis in 2011, with the number increasing annually. Among them, a large number of migrant workers suffering from pneumoconiosis were not medically diagnosed. Therefore, fundamental questions remain unanswered: what is the background of workers who receive a diagnosis of pneumoconiosis, and how does pneumoconiosis affect their future and well-being?

**Methods:**

In this study, we identified and surveyed 1,134 workers with pneumoconiosis in seven selected regions in China with substantially high incidences of pneumoconiosis by using a combination of cluster sampling, convenience sampling, and snowball sampling. We used demographic, medical, and rehabilitation conditions and welfare questionnaires to collect the data.

**Results:**

The findings highlighted the socioeconomic status of patients with pneumoconiosis. The majority of workers with pneumoconiosis were adult men who had received no higher education, who lived in rural households, and who were employed in mining or manufacturing industries. Among these workers, 52.8% had been exposed to dust at work for more than 10 years, and 53.1% received a diagnosis of stage II or III pneumoconiosis. More than half of the workers (569 workers, 50.2%) did not receive comprehensive, routine treatment; 33.4% (379 workers) visited a doctor when they experienced physical discomfort, and 6.6% (75 workers) never received treatment. Only 156 workers (13.8%) received rehabilitation services, whereas 978 workers (86.2%) never did. The study results also revealed the severe financial difficulties faced by patients with pneumoconiosis. Only 208 workers (18.3%) had access to work-related injury insurance, with the cost of pneumoconiosis treatment being a substantial burden for 668 workers (60.6%).

**Conclusion:**

In this study, we explored the existing health and welfare problems faced by workers with pneumoconiosis in China and identified the social injustice and health disparities that these workers experience. We also clarified the primary challenges in implementing safety, health, and welfare policies for these workers and those who are exposed to high-risk environments, such as those working in mining.

## Introduction

With the progress in industrial development among societies worldwide, several large, costly, and dangerous industrial structures have been observed. This is why improving public health and safety has been a concern for governments worldwide, because it is not only a problem of environmental justice but also a matter of fairness, with health departments and workers struggling with occupational hazards and environmental risks (1, 2). Hence, occupational injuries must be considered when discussing sustainable development, which is concerned with the compensation, well-being, and livelihood of workers operating in high-risk environments (3, 4).

Pneumoconiosis, a preventable lung disease caused by occupational exposure to dust, is one of the most severe occupational hazards worldwide (5). Multiple country-specific studies on pneumoconiosis have emphasized the risks and severity of this disease (6–9). However, the incidence of occupational lung disease is consistently underreported worldwide. This is because of the difficulty of identifying work-related etiologies and physicians’ insufficient awareness of occupational respiratory carcinogens in many developed European nations (10). Compared with other countries, developing countries have more limitations regarding pneumoconiosis diagnosis, occupational hazard awareness, comprehensive national legislation, occupational health and safety policy, and risk-based occupational medical examinations for workers (11).

In the 1960s, when a single silicosis case was officially identified as an occupational lung disease in China, pneumoconiosis gained widespread attention (12). In 1978, in response to the rapid expansion of rural township enterprises and small and medium-sized businesses and changes in labor laws, industry relations and economic development reforms were introduced. Since then, the production environment of small coal mines and small factories in a number of towns has been harsh, and the problem of dust pollution has become severe (13). According to the Ministry of Health’s report on occupational diseases, by the end of 2000, 558,624 cases of pneumoconiosis had been reported. Generally, pneumoconiosis is prevalent in the mining, metal casting, machine manufacturing, construction, blasting, and excavation industries. According to the geographic distribution of the disease, the provinces of Sichuan, Hunan, Liaoning, Shanxi, Jiangxi, and Heilongjiang seem to be disease hotspots in China. New cases of pneumoconiosis in mainland China have been characterized by short working years and severe illness, especially among small and medium-sized businesses. Many migrant workers who returned to their hometowns have received a diagnosis of pneumoconiosis. By the end of 2011, China had 676,541 officially reported cases of pneumoconiosis; however, an estimated six million migrant workers had unofficial cases of pneumoconiosis (14), as confirmed by several private agencies, including Love Save Pneumoconiosis and China Labour Bulletin (15).

China has attempted to develop solutions for occupational diseases on the basis of its past experiences and the experiences of other nations (16–18). Recent studies have focused on the pathology of pneumoconiosis and on prevention strategies, methods, and techniques (12, 18, 19). In addition, empirical studies based on epidemiology have been conducted on collieries and mining groups in specific regions of China to examine the prevalent characteristics of pneumoconiosis, such as the regional distribution, enterprise type, trends, life expectancy, and mortality rate (19–23).

As an occupational disease, debilitating and irreversible pneumoconiosis injuries can have devastating social and societal consequences. However, occupational safety has not been adequately discussed in China. Although research on pneumoconiosis and patients with this disease has aided in the promotion of occupational health awareness, fundamental questions remain unanswered: what is the background of workers who receive a diagnosis of pneumoconiosis? Do they experience health disparities? How does pneumoconiosis affect their future and well-being? In this study, we focused on the individual characteristics, current status, and welfare problems of workers with pneumoconiosis. Generally, public officials and scholars may not be aware of workers with pneumoconiosis and their life conditions and well-being after they contract pneumoconiosis. In addition, to the best of our knowledge, no social policy analysis surveys have been conducted to examine the actual lives of workers with pneumoconiosis. Therefore, in this study, we provided evidence to fill the current gap in the literature. We examined 1,134 cases of pneumoconiosis from seven regions of China. We attempted to overcome data collection challenges; illustrate the main characteristics, current medical treatment and rehabilitation conditions, and difficulties of workers with pneumoconiosis; and provide evidence for the future planning of pneumoconiosis policy and welfare.

## Methods

From June 2014 to January 2016, we conducted a questionnaire survey in seven regions of China, with patients with pneumoconiosis as the sampling population and patients with pneumoconiosis and their families as the sampling units. The participants received either a clinical diagnosis at a medical institution or a diagnosis of occupational disease at a specialized agency or had symptoms or a history of occupational exposure.

Studying the available literature and media reports (19, 24–26) revealed a connection between the geographic distribution of pneumoconiosis incidence and pneumoconiosis propensity. On the basis of existing literature and expert consultation, districts with the highest prevalence of pneumoconiosis are typically those represented by the usual industrial clusters and with a high labor export rate. Therefore, judgment sampling of the survey was first used. Because of the disparity in economic growth among Chinese provinces, a few representative provinces were initially selected depending on their developmental status and labor mobility across regions; that is, seven survey areas were selected as the sampling frame. To collect data from each selected region, a combination of cluster sampling, convenience sampling, and snowball sampling was used. In other words, in areas where the number of patients with pneumoconiosis was less than 200, cluster sampling was used to select respondents. By contrast, in areas where the number of patients with pneumoconiosis exceeded 200, convenience sampling and snowball sampling were used to select respondents. Subsequently, on the basis of the preliminary survey findings, six of these provinces, namely those with a large number of patients with pneumoconiosis, were selected. These provinces represented typical industrial clusters with a high prevalence of pneumoconiosis and regions with a high labor export rate. Statistically, if more than 200 samples were collected in each region, they could largely represent the status of workers with pneumoconiosis in the province. We ultimately distributed 1,200 surveys across seven provinces; Shaanxi was eventually included in the practical survey, thereby meeting the sample requirements of a 95% confidence interval and a 3% margin of error (statistically, the number of survey samples should reach at least 1,067). Table 1 shows the final geographic distribution of pneumoconiosis cases across the seven regions.

**Table 1:** Locations, periods, and sample sizes of the survey.

Geographical area	Location	Period	Total (Valid)
Eastern China	Fangshan District, Beijing	2016.01	202 (202)
Northeast China	Chaoyang City, Liaoning Province	2014.06	238 (238)
Central China	Shiyan City, Hubei Province	2015.09–2016.01	178 (178)
	Jiujiang City, Jiangxi Province	2015.01	185 (185)
	Lu’an City, Anhui Province	2015.07	182 (126)
Western China	Wuwei City, Gansu Province	2014.08	52 (52)
	Ankang City, Shanxi Province	2014.08	153 (153)
Total			1,190 (1,134)

The survey questionnaire was adapted from the Chinese version of the World Health Organization’s Instrument for Measuring Quality of Life (WHOQOL-100) and St. George’s Respiratory Questionnaire and included questions regarding the demographic background, socioeconomic status, medical conditions, rehabilitation conditions, medical expense burden, and welfare conditions of workers with pneumoconiosis. Before completing the questionnaire, Respondents were informed that their responses would be kept anonymous and confidential, and they signed an informed consent form.

A questionnaire was deemed invalid if it met three basic criteria: (1) at least 10% of the questions were left unanswered or multiple responses were selected, (2) at least 3% of the questions had contradictory responses, and (3) at least 50% of the questions in two surveys had the same responses. Any questionnaire meeting one of these three criteria was considered invalid. In this study, 1,200 questionnaires were distributed, and 1,190 responses were received, of which 1,134 were valid. Because more than 95% of the questionnaire responses were valid, the survey was considered reliable (the actual rate was 95.3%).

## Results

### Basic characteristics

Workers with pneumoconiosis are typically employed in industries that expose them to dust. Our findings revealed that pneumoconiosis was predominantly observed in men involved in the mining industry. The study participants (*N* = 1,134) included a total of 1,123 male workers. Among workers with pneumoconiosis, 869 (76.6%) were under 60 years of age, 352 (31%) were 51–60 years of age, 415 (36.6%) were 41–50 years of age (the highest proportion), and 98 (8.6%) were 31–40 years of age. The median age of the patients was approximately 52 years. In total, 891 workers (78.6%) were registered in a rural *Hukou*,^1^ and 889 workers (78.4%) resided in rural areas at the time of the study. Educational background statistics revealed that workers with pneumoconiosis had not received higher education. A total of 617 workers (54.4%) had completed primary school, and 447 workers (39.4%) had completed junior high school. In terms of marital status, 95.4% of those surveyed reported being married.

The majority of workers with pneumoconiosis were employed in the mining or manufacturing industries and were rural migrant workers with poor technical skills. According to our findings, 1,016 workers (89.6%) were engaged in mining before they received a diagnosis of pneumoconiosis. We also investigated the exposure years of workers with pneumoconiosis. Our results also indicated that 210 workers (18.5%) were exposed to dust for less than 5 years, 325 workers (28.7%) were exposed to dust for 5–10 years, and 599 workers (52.8%) were exposed to dust for more than 10 years. Regarding the current employment status, 464 workers (40.9%) reported being unable to work, 224 workers (approximately 20%) reported a limited capability to return to agricultural work, 29 workers (approximately 2.6%) reported a partial incapacity and inability to find suitable jobs, and 197 workers (17.4%) with reduced physical strength reported working in nonagricultural fields, such as in security and delivery services (see Table 2).

**Table 2:** Characteristics of study participants.

Statistic characteristic		Cases (%)
Gender	Male	1,123 (99)
	Female	11 (1)
Age	Under 30 years old	4 (0.4)
	31–40 years old	98 (8.6)
	41–50 years old	415 (36.6)
	51–60 years old	352 (31)
	Over 60 years old	265 (23.4)
*Hukou* type (Household registration)	Urban *Hukou*	243 (21.4)
	Rural *Hukou*	891 (78.6)
Current residence area	Urban area	245 (21.6)
	Rural area	889 (78.4)
Education level	Primary school and below	617 (54.4)
	Junior school	447 (39.4)
	High school	61 (5.4)
	College education or above	9 (0.8)
Marital status	Married	1,082 (95.4)
	Single, divorced or widowed	52 (4.6)
Occupational/Industry distribution before being diagnosed	Mining	1,016 (89.6)
	Machine Manufacture Industry	53 (4.7)
	Construction Industry	23 (2)
	Metal casting	11 (1)
	Blasting, Excavating	11 (1)
Years of exposing to dust dusty work	Below 5 years	210 (18.5)
	5–10 years	325 (28.7)
	10–15 years	217 (19.1)
	15–20 years	122 (10.8)
	Over 20 years	260 (22.9)
Current employment status	Non-agricultural work	197 (17.4)
	Have work ability but unemployed	29 (2.6)
	Agricultural work	224 (19.8)
	Work inability	464 (40.9)
	Retired or other situations	220 (19.4)

### Diagnosis and treatment characteristics

In our study, silicosis cases comprised the largest proportion of all pneumoconiosis types (997 workers, 87.92%). The cases of pneumoconiosis included coal workers’ pneumoconiosis (CWP) (6.61%), graphite pneumoconiosis (0.35%), and other types of pneumoconiosis (see Table 3). Silicosis and CWP constituted 94.5% of all types of pneumoconiosis. Pneumoconiosis is a severe lung disease that causes permanent damage to the lungs and heart and is sometimes fatal. According to the National Pneumoconiosis Diagnostic Criteria of China, pneumoconiosis is divided into three categories depending on size, profusion, and distribution range of opacities, namely stages I, II, and III. When the disease advances from stage I to stage II and then to stage III, patients lose their strength and eventually their ability to work. In this study, 326 workers (28.7%) had stage I pneumoconiosis, 403 workers (35.5%) had stage II pneumoconiosis, 200 workers (17.6%) had stage III pneumoconiosis, and 205 workers (18.1%) were unaware of their pneumoconiosis stage. When asked about the progression of their pneumoconiosis status, 58% of the participants reported no aggravation of their pneumoconiosis status, 21.4% reported aggravation of their pneumoconiosis status, and nearly 23.5% (233 workers) lacked adequate knowledge of their illness (i.e., they did not know whether their disease had progressed), indicating that they had faced difficulties in disease examination and diagnosis. More than 65% of the participants reported that their pneumoconiosis had lasted more than 4 years.

**Table 3:** Diagnostic and treatment characteristics of study participants.

Diagnostic and treatment characteristics		Cases (%)
Categories of pneumoconiosis	Silicosis	997 (87.9)
	CWP	75 (6.6)
	mica	18 (1.6)
	cement	12 (1.1)
	welding workers’	7 (0.6)
	graphite	4 (0.4)
	talc	4 (0.4)
	caster	4 (0.4)
	carbon black	3 (0.3)
	asbestos	2 (0.2)
	Others	8 (0.7)
Pneumoconiosis stages	stage I	326 (28.7)
	stage I	403 (35.5)
	stage III	200 (17.6)
	Not clear about the stage	205 (18.1)
Progression of pneumoconiosis status	No aggravation of pneumoconiosis status	658 (58)
	Aggravation of pneumoconiosis stage: from stage I to stage II	139 (12.3)
	Aggravation of pneumoconiosis stage: from stage II to stage III	65 (5.7)
	Aggravation of pneumoconiosis stage: from stage I to stage III	39 (3.4)
	Not sure whether pneumoconiosis status	233 (23.5)
Duration of pneumoconiosis	Under 1 year	105 (9.3)
	1–3 years	279 (24.6)
	4–6 years	297 (26.2)
	Over 6 years	453 (39.9)
Comprehensive routine treatment since diagnosis	YES	565 (49.8)
	No	569(50.2)
Seeking medical care without comprehensive routine treatment	Visiting the doctor actively	115 (10.1)
	Visit a doctor when experiencing physical discomfort	379 (33.4)
	Never receive treatment	75 (6.6)
Medical institutions selected	Village and/Community clinics	181 (16)
	Town-level health centers	444 (39.2)
	County-level hospitals	267 (23.5)
	Municipal or higher-level hospitals	155 (13.6)
	Others	87 (7.7)

More than half of the workers (569 workers, 50.2%) reported receiving no comprehensive routine treatment after they had received their diagnoses; 33.4% (379 workers) visited a doctor when they experienced physical discomfort, and 6.6% (75 workers) never received treatment. Regarding the selection of medical institutions for treatment, the following trends were observed: 182 workers (16%) visited village or community clinics, 444 workers (39.2%) visited town-level health centers, 266 workers (23.5%) visited county-level hospitals, and 154 workers (13.6%) visited municipal or higher-level hospitals.

### Rehabilitation conditions

Our findings indicated that pneumoconiosis rehabilitation services are not easily accessible. According to our study, only 156 workers (13.8%) received rehabilitation services, whereas 978 workers (86.2%) never did (see Table 4). Regarding rehabilitation content, health education was the most commonly provided (78.8%), including diet counseling and lifestyle modifications. In addition, 55.8% of the participants received respiratory training, 42.9% received expectoration training, 41% received oxygen training, 37.8% received psychological counseling, and 16% received full-body aerobic exercise training, including instruction in hiking and cycling. However, not all workers who received rehabilitation services received them from professionals. Some workers (17.3%) had limited knowledge about rehabilitation and chose to handle their own therapy by communicating with other patients with pneumoconiosis (23.7%) or with their family members (12.8%). The typical length of a rehabilitation program ranges from a few weeks to 3 years. The American College of Chest Physicians recommends 12 weeks for rehabilitation programs (27). In this study, 66% of the workers received rehabilitation services once every 2 months and even sometimes after 2 months, and only 18.6% received rehabilitation services once a week or more. These percentages indicate that additional efforts are required to improve the quality of rehabilitation services.

**Table 4:** Rehabilitation conditions of study participants.

Rehabilitation conditions		Cases (%)
Rehabilitation since diagnosis	YES	156 (13.8)
	No	978 (86.2)
Rehabilitation content	Health education	123 (78.8)
	respiratory training	87 (55.8)
	Expectoration training	67 (42.9)
	Oxygen training	64 (41)
	psychological counseling	57 (37.8)
	Full-body aerobic exercise training	25 (16)
Source(s) of rehabilitation service or knowledge	Patients themselves	27 (17.3)
	Professionals	125 (80.1)
	Other patients	37 (23.7)
	Family members	20 (12.8)
Frequency of rehabilitation services	once a week at least	29 (18.6)
	once every two months at least	103 (66)
	occasionally	24 (15.4)

### Welfare and medical burden

The social security program is intended to play a crucial role for workers who cannot maintain a minimum living standard when they have lost their ability to work or encounter unforeseen natural disasters beyond their control. Work-related injury insurance (WII) is designed to provide workers with pneumoconiosis with financial security. In our study, we asked the participants about the social protection program benefits that they had received. Only 208 workers (18.3%) had access to WII, 421 workers (37.2%) received the guaranteed minimum income, and 231 workers (20.4%) received other assistance or relief from social organizations, such as charity organizations.

Workers with pneumoconiosis are typically the primary breadwinners in their families, and their inability to work coupled with high medical expenses traps their families in a cycle of poverty. Most patients with pneumoconiosis without WII coverage are required to pay out of pocket (OOP) for treatment. Therefore, in this study, we collected information regarding the OOP expenses of patients with pneumoconiosis. At the time of investigation, the average monthly household income of the majority of workers with pneumoconiosis (661 workers, 58.5%) was less than 2000 RMB. The average monthly household income was approximately 1945 RMB. However, the annual average cost of treatment was as much as 6,964 RMB. Self-rating of economic status revealed that 375 workers (33.1%) were impoverished and frequently in debt and that 462 workers (40.7%) had relatively weak family financial status. Similarly, self-rating of financial burden revealed that the medical treatment cost for pneumoconiosis was a substantial burden for 668 workers (60.6%) because they and their families could not afford or could barely afford the medical expenses (see Table 5).

**Table 5:** Welfare and self-rated economic and medical treatment cost burden characteristics of study participants.

Category	Information	Cases (%)
Social protection programs benefits being received	basic health insurance for residents	837 (73.8)
	basic health insurance for workers	222 (19.6)
	basic pension for residents	101 (8.9)
	basic pension for workers	265 (23.4)
	work-related injury insurance	208 (18.3)
	guaranteed minimum income	421 (37.2)
	Others assistance or relief	231 (20.4)
	none	17 (1.50)
Monthly household income (RMB)	Less than 1,500	417 (36.8)
	1,501–2000	244 (21.5)
	2001–3,000	246 (21.7)
	More than 3,001	221 (20)
Average Monthly household income (RMB)		1945.642
Annual cost of treatment (RMB)	Less than 3,600	449 (39.6)
	3,601–7,200	261 (23)
	7,201–12,000	144 (12.7)
	12,001–18,000	99 (8.7)
	More than 18,001	106 (9.3)
	(Missing value)	75 (6.6)
Average Annual cost of treatment (RMB)		6964.027
Self-rating of economic status	Quite good	18 (1.6)
	Average, but meets basic expenses	279 (24.6)
	Quite poor, needs careful calculation	462 (40.7)
	Very poor, often in debt	375 (33.1)
Self-rating of medical treatment cost	Very heavy	268 (25.3)
	Relatively heavy	400 (35.3)
	Not very heavy	278 (24.5)
	Not heavy	113 (10)
	(Missing Value)	75 (6.6)

## Discussion

Our findings revealed that the majority of workers with pneumoconiosis were adult men, which is in line with previous research indicating that men make up the majority of individuals with pneumoconiosis in China (28). These patients typically have low levels of education, reside in rural households, and work in the mining or manufacturing industries. This is confirmed by the high incidence of pneumoconiosis among Chinese coal miners (18). Occupational diseases progress rather slowly (i.e., over months or even years), although workers may have been exposed to toxic materials for many years without exhibiting any effects (29). Most of the participants included in this study were exposed to dust for more than 5 years and had stage II or III pneumoconiosis, which is a disabling chronic illness that is difficult to treat. Patients with pneumoconiosis typically experience reduced lung function (30), mood changes, respiratory symptoms, and decreased physical exercise tolerance. According to our findings, more than 40% of the participants lost their ability to work, indicating the difficulty that patients with pneumoconiosis experience while attempting to engage in productive work. The majority of these migrant workers were forced to return to their hometowns after receiving their diagnoses because of their diminished work capacity and a lack of fundamental labor rights and workplace safety. This in turn had a negative effect on their quality of life.

Our findings revealed that the majority of patients received inadequate or delayed treatment and rehabilitation options under the current standard of care, with a lack of occupational health services and medical care. Most of these patients (78.4%) lived in rural areas with limited health and medical services; medical institutions that are capable of providing targeted treatment are primarily distributed across municipalities. Therefore, traveling to the city to seek treatment entailed expenses associated with diagnosis, outpatient or inpatient treatment, transportation, food, and lodging. This study has highlighted the unequal distribution of resources across regions and that access to occupational and medical health institutions is one of the most critical concerns for patients with pneumoconiosis. In addition to treatment and medication, rehabilitation is vital for patients with pneumoconiosis. Pulmonary rehabilitation is frequently used to alleviate the symptoms of pneumoconiosis and improve the patients’ exercise capacity, mood, health-related quality of life, and longevity. Although the content and settings of rehabilitation vary from one occupational health institution to another, they primarily cover moderate physical training, health education, psychological counseling, and nutritional counseling (31, 32). Our findings, however, revealed that the majority of patients did not have access to high-quality, comprehensive rehabilitation systems.

Deteriorating labor capacity worsened upon failing to work because of sickness, and worst of all, it was also revealed that most workers with pneumoconiosis lacked WII (compensation system) coverage. WII covers the expenses of medical treatment, including clinical procedures, pharmaceutical care, and hospitalization for work-related injuries, as well as the costs associated with the accommodation and transportation of those seeking medical treatment outside their home areas. This is why the lack of a work-related injury compensation system is a serious concern for patients with pneumoconiosis. In situations where OOP medical expenses are high in the absence of WII coverage, the heavy burden of medical treatment costs creates a problem for workers with pneumoconiosis and their families. As the primary breadwinners in the families, many workers with pneumoconiosis who participated in the study were compelled to forsake treatment in order to protect their families from further economic hardship.

The primary process for workers with pneumoconiosis to receive compensation is on the basis of a diagnosis of pneumoconiosis. In this study, 925 patients (81.3%) with pneumoconiosis did not receive a medical certificate of specialized occupational disease. If workers with pneumoconiosis do not receive a diagnosis, they do not qualify for compensation. Several authoritative and legitimate occupational disease evaluation centers are available at different levels (province, municipality, and county) to diagnose pneumoconiosis and related occupational diseases.^2^ In this study, we discovered that the majority of workers with pneumoconiosis were migrant workers. Some of these workers returned to their hometowns to receive their diagnoses from a local occupational disease center. However, these diagnoses were rarely recognized by local officials at their work locations. Therefore, after receiving a diagnosis of pneumoconiosis, the majority of workers with this severe occupational disease were unable to obtain any compensation or benefits. This is one of the reasons why the current official statistics of pneumoconiosis are underestimated, as they are strictly based on the diagnosis and identification of occupational diseases.^3^

By investigating the socioeconomic status, clinical characteristics of pneumoconiosis, follow-up treatment and rehabilitation, economic burden, and social protection status of patients with pneumoconiosis, our study revealed that these patients may have psychological well-being problems, which is an issue that has been neglected in recent studies (33). This suggests that we should pay more attention not only to pneumoconiosis from a social and economic perspective but also from the psychological well-being perspective, as the disease substantially affects patients’ and their families’ quality of life. In the follow-up research, we will focus on pneumoconiosis patients’ quality of life and mental health.

## Conclusion

This study attempts to overcome challenges relating to data collection and ultimately provides empirical evidence for how the socioeconomic status of people with pneumoconiosis is linked to their clinical characteristics, current medical treatment and rehabilitation conditions, and difficulties faced by workers with pneumoconiosis. In this study, we explored the existing health and welfare problems faced by workers with pneumoconiosis in China and highlighted the social injustice and health disparities that these workers experience. We also identified the main challenges in implementing safety, health, and welfare policies for these workers and those operating in high-risk environments.

According to our findings, there is a pressing need to bolster efforts in supporting social sustainability and ensuring the health and welfare rights of workers with pneumoconiosis. A closer examination reveals potential areas of improvement in recognizing the predominant characteristics and needs of these workers. To effectively address the medical and health issues of workers with pneumoconiosis, it is crucial to pool all available resources in occupational health. The primary tenets of pneumoconiosis care should encapsulate availability, acceptability, accessibility, and affordability. It is also essential to raise awareness among affected workers about how rehabilitation therapy might enhance their quality of life.

For enhanced policy implementation and to furnish comprehensive assistance to individuals with pneumoconiosis, both systemic and procedural barriers, such as district limitations, time constraints, and nuances of informal employment relationships, should be reconsidered. This might streamline the process of ascertaining the occupational origin of the disease. It’s paramount to put in place legal frameworks that pave the way for compensation and assistance, as this can significantly contribute to the evolution of the social security system and the betterment of the health status of workers with pneumoconiosis. Moreover, improving all dust-exposed workplace environments is a necessity, coupled with robust efforts to mitigate the disease’s onset. Prevention stands at the forefront for conditions like pneumoconiosis, given its incurable nature. Instituting and adhering to safety protocols in workplaces can curtail the exposure of workers to dust pollutants. Implementing proactive preventive measures can not only decrease the incidence of pneumoconiosis but also further the aims of public health and social sustainability.

In response to the pneumoconiosis issue, several strategic interventions merit consideration. Primarily, stringent adherence to the “Law of the People’s Republic of China on the Prevention and Control of Occupational Diseases” and the “Law of the People’s Republic of China on Work Safety” could potentially enhance pneumoconiosis prevention and treatment efforts. The oversight of relevant government departments could ensure employers’ compliance with these laws, thereby ensuring legal labor relationships and appropriate insurance coverage. Reviewing the procedures for occupational disease identification could offer significant benefits. The empowerment of county-level and above hospitals to carry out occupational disease identification may disrupt existing monopolies and promote a more inclusive and comprehensive system. The promotion of industrial trade unions could foster the formulation of industry-wide occupational safety protection standards. Such standards, developed through collective bargaining, could encourage technological upgrades and improved labor protection measures in enterprises. The establishment of a national special relief fund for pneumoconiosis could provide much-needed support for affected individuals. Such a fund might also prompt responsible companies to be held accountable, thereby reinforcing corporate social responsibility. The implementation of community reconstruction plans in pneumoconiosis-stricken areas could be advantageous. Given the collective impact of this disease on families and communities, efficient interventions targeting family relief, child counseling, elder support, and community rebuilding merit consideration. The involvement of existing social work institutions in urban areas, facilitated by policy promotion, financial contribution, and active implementation of community reconstruction programs, could yield substantial improvements.

This study has some limitations. The survey sample may not be fully representative of the pneumoconiosis situation in China because it was selected using judgmental sampling based on a literature review and expert judgment. In addition, to study pneumoconiosis, an in-depth examination of its epidemiological characteristics is required, a weakness that this study highlights.

## Data availability statement

The raw data supporting the conclusions of this article will be made available by the authors, without undue reservation.

## Author contributions

WH conducted the analysis, drafted the original manuscript, and contributed to its revision. W-NW contributed to the drafting of the manuscript and critically revised it. QQ designed and supervised the study. All authors contributed to the article and approved the submitted version.

## Conflict of interest

The authors declare that the research was conducted in the absence of any commercial or financial relationships that could be construed as a potential conflict of interest.

## Publisher’s note

All claims expressed in this article are solely those of the authors and do not necessarily represent those of their affiliated organizations, or those of the publisher, the editors and the reviewers. Any product that may be evaluated in this article, or claim that may be made by its manufacturer, is not guaranteed or endorsed by the publisher.

## Footnotes

^1^The household registration system in China, which classifies each individual as a rural or urban resident, is a major means of regulating population mobility and determining eligibility for state-provided services and welfare.

^2^Both the Law on Prevention and Treatment of Occupational Diseases and Management Regulations for Diagnosis and Assessment of Occupational Diseases state that workers are free to choose whether to have their occupational injury or illness diagnosed either at their local occupational disease center or at their employer’s location.

^3^Alternatively, the cases of pneumoconiosis recorded in official reports only document workers at sizeable state-owned coal mines, as well as in the metal casting, construction, mining, chemical, and other industries. The excluded data may be from informal employment relationships and local and township coal mines.
